# Role of Diagnostic Imaging in Chronic Recurrent Multifocal Osteomyelitis (CRMO) in Children: An Observational Study

**DOI:** 10.3390/children8090792

**Published:** 2021-09-10

**Authors:** Michał Kopeć, Magdalena Brąszewska, Mariusz Jarosz, Katarzyna Dylewska, Andrzej Kurylak

**Affiliations:** 1 Department of Imaging Diagnostics, Regional Children Hospital in Bydgoszcz, 85-667 Bydgoszcz, Poland; 2 Department of Pediatrics, Hematology, Oncology and Rheumatology, Regional Children Hospital in Bydgoszcz, 85-667 Bydgoszcz, Poland; madziorekm@op.pl (M.B.); mariusz.jarosz1987@gmail.com (M.J.); k.dylewska16@gmail.com (K.D.); andrzej.kurylak@wsd.org.pl (A.K.); 3 Department of Preventive Nursing, The Nicolaus Copernicus University, Collegium Medicum in Bydgoszcz, 85-821 Bydgoszcz, Poland

**Keywords:** chronic recurrent multifocal osteomyelitis, magnetic resonance, diagnostic imaging

## Abstract

The aim of this single-center observational study was to analyze the applicability of various imaging studies to the diagnosis and further evaluation of patients with chronic recurrent multifocal osteomyelitis (CRMO). The analysis included the data of 10 patients with CRMO treated between 2016 and 2021. The mean ages of the patients at the first manifestation of CRMO and ultimate diagnosis were 10 years and 7 months and 11 years and 10 months, respectively. Conventional radiography demonstrated focal loss of bone density in only 30% of the patients. Computed tomography showed disseminated foci with non-homogeneous osteolytic/osteosclerotic structure, with a massive loss of cortical layer and strong periosteal reaction. On magnetic resonance imaging (MRI), most patients presented with multifocal hypodense areas on T1-weighted images, with the enhancement of signal on T-weighted and STIR sequences. The duration of follow-up varied between 3 months and 3 years. In 40% of the patients, both clinical symptoms and the abnormalities seen on MRI resolved completely, whereas another 50% showed partial regression of clinical and radiological manifestations. MRI findings, co-existing with characteristic clinical manifestations, play a pivotal role in establishing the ultimate diagnosis of CRMO. MRI can also be used to monitor the outcomes of treatment in CRMO patients.

## 1. Introduction

Chronic recurrent multifocal osteomyelitis (CRMO) is a rare form of non-infectious inflammation of the bones in children and young adults. The disease was first described by Giedon et al. in 1972 as a “syndrome of subacute, chronic and symmetric bone lesions” [1]. According to European registries, CRMO represents 2–5% of all cases of osteomyelitis, and its incidence does not vary geographically. The prevalence of CRMO among children is estimated at 1 per 160,000 to 1 per 2,000,000 [2]. Initial manifestations of the disease are typically observed between 7 and 12 years of age, with a median age at the onset of 10 years, and a four to one female to male patient ratio [2]. Although the first case of the disease was described nearly 50 years ago, the etiology of CRMO is still unknown. However, co-occurrence of CRMO with autoimmune disorders, such as inflammatory bowel disease, coeliac disease and psoriasis, implies that the disease is associated with a systemic inflammation with a disrupted balance between anti-inflammatory and proinflammatory cytokines and resultant immune deregulation. Research on the etiopathogenesis of CRMO included various cytokines, such as interleukins (IL), IL-1, IL-6 and IL-10, and, in particular, tumor necrosis factor (TNF) alpha, known for its pro-apoptotic effect on osteoclasts [3,4]. It is likely that the etiopathogenesis of CRMO also includes a genetic predisposition [5]. In children with Majeed syndrome, associated with a mutation of the LPIN2 gene on chromosome 18p, and patients with the deficiency of IL-1 receptor antagonist caused by a mutation of the IL1RN (DIRA) gene, the signs of non-bacterial osteomyelitis appear already in early childhood [3]. CRMO is a pediatric variant of SAPHO (synovitis, acne, pustulosis, hyperostosis, osteitis) syndrome in adults, described first by Chamot in 1987.

The first manifestation of CRMO is transient pain in the affected bone or adjacent joint. Usually, the symptoms exacerbate at night causing awakening and can be attenuated with non-steroid anti-inflammatory drugs. Rarer symptoms of the disease include swelling or redness with increased temperature of the skin. In 25% of patients, CRMO may be associated with skin lesions (acne, psoriasis, palmoplantar pustulosis), low-grade fever and loss of weight. The disease can involve any bone, and the lesions are usually symmetrical. Unifocal CRMO is found rarely, typically in the epiphyses of long bones, such as the femur, tibia and fibula (34%) or the clavicles (24%) and can be misdiagnosed as a neoplastic process [6]. Other, less frequent locations of CRMO include vertebral bodies, and the mandible and pelvic bones [7].

No specific diagnostic markers of CRMO have been identified thus far. Laboratory tests play merely an adjunct role. Inflammatory markers are usually within respective normal limits or slightly elevated. Additionally, the levels of rheumatoid factor and autoantibodies specific for systemic connective tissue disorders are normal, and no link has been found between CRMO and the HLA B27 antigen. The results of microbiological and serological tests for bacterial, viral and fungal infections are negative. Histopathological examination shows non-specific subacute or chronic osteomyelitis [5,8], with the infiltration of bones with neutrophils, plasma cells, lymphocytes, macrophages and histiocytes.

Imaging studies are vital for the diagnosis of CRMO. Bone pathologies may not be visualized at the early stages of the disease. During the first phase of CRMO, imaging findings include osteolysis, followed by bone sclerosis and bone remodeling. Another characteristic feature of the disease are periosteal reactions. The presence of osteolytic foci may raise a suspicion of a neoplastic process.

While the role of computed tomography (CT) in the diagnosis of CRMO is limited, CT can visualize small bone lesions within the sternum, spine and pelvis. Given the lack of exposure to ionizing radiation, the safest and, simultaneously, the most effective diagnostic imaging modality in detecting CRMO, monitoring its outcomes and treatment effectiveness is magnetic resonance imaging (MRI). Together with technetium scintigraphy, MRI can detect latent, asymptomatic lesions [3].

Despite many attempts, no unified diagnostic criteria of CRMO have been defined thus far. The most widely accepted diagnostic criteria were proposed by Jansson et al. [8] and Roderick et al. [9]. The algorithm proposed by Jansson et al. [8] includes four major (radiologically proven osteolytic/sclerotic bone lesions, multifocal bone lesions, palmoplantar pustulosis or psoriasis (PPP), sterile bone biopsy with signs of inflammation and/or fibrosis, sclerosis) and six minor criteria (normal blood count and good general state of health, C-reactive protein (CRP) and erythrocyte sedimentation rate (ESR) mildly-to-moderately elevated, observation time > 6 months, hyperostosis, association with other autoimmune diseases apart from PPP and psoriasis, and grade I or II relatives with autoimmune or autoinflammatory disease). CRMO is diagnosed whenever a patient satisfies at least two major criteria or at least one major and three minor criteria [8]. Alternatively, a diagnostic score can be calculated based on the observation that CRMO patients typically present with low levels of inflammatory markers, normal body temperature and bone lesions in typical locations. The patient is likely to suffer from CRMO if the overall score is 39 points or higher (Table 1) [10].

In 2016, Roderick et al. [9] proposed the so-called Boston diagnostic criteria based on clinical manifestation, radiological findings, the location of bone lesions and the results of laboratory tests. Patients who satisfy four criteria (typical clinical symptoms, typical imaging findings, multifocal lesions or unifocal lesion within the clavicle and CRP < 30 g/L) are likely to present with CRMO, whereas in those satisfying the first two criteria but presenting with unifocal extraclavicular lesions and elevated CRP, the diagnosis should be confirmed by bone biopsy (plasma cell infiltration, fibrosis, sclerotization and negative result of microbiological testing).

Another algorithm facilitating the qualification of patients with suspected CRMO to require a bone biopsy was proposed by Taddio et al. [11]. According to this algorithm, the biopsy is advisable in patients in poor general condition, with elevated inflammatory markers, abnormal complete blood counts, non-specific imaging findings or unifocal disease. The decision to perform a biopsy should be made on an individual basis after carefully weighing all potential benefits and risks.

Given the non-specific course of the disease, CRMO is a diagnosis of exclusion. In patients with fever, elevated inflammatory markers and poor general condition, the primary diagnosis is infectious osteomyelitis. The unifocal character of the disease with the evidence of osteolysis on imaging studies raises suspicion of primary bone malignancy (osteosarcoma), especially if the lesions are located in the epiphyses of long bones. Skin manifestations and concomitant nail psoriasis warrant evaluation for psoriatic arthritis as a cause of bone/joint pain. Pain in the lumbosacral spine may be a manifestation of a spondyloarthropathy.

CRMO is characterized by periodical exacerbations and remissions, and, hence, the identification of patients in whom the disease will resolve spontaneously and those who require intensive long-term anti-inflammatory treatment can be challenging. The first line of treatment includes non-steroid anti-inflammatory drugs (NSAIDs). In 80% of the patients, this treatment results in complete or partial clinical and radiological remission within 6 months [10]. According to the consensus statement from 2018, patients who do not respond adequately to the NSAID therapy may additionally receive glucocorticoids (prednisone with the initial dose of 2 mg/kg/day, no more than 60 mg/day, followed by the maintenance dose of 0.1–0.2 mg/kg/day) with the evaluation of response after 6 weeks. In 65% of the patients, the treatment results in remission within 3 to 6 months [12,13]. Whenever the activity of the disease is high, the patients may be switched to disease-modifying antirheumatic drugs (DMARDs, usually methotrexate or sulfasalazine), TNF inhibitors or bisphosphonates. The therapy should be continued for at least 12 months [14], except for bisphosphonates, which should be administered for 3–6 months [15].

The aim of this single-center observational study was to analyze the applicability of various imaging studies to the diagnosis and further evaluation of patients with CRMO.

## 2. Materials and Methods

The study included 10 patients, 7 girls and 3 boys, treated at the Department of Pediatrics, Hematology, Oncology and Rheumatology of the Regional Children Hospital in Bydgoszcz between 2016 and 2021.

The patients were referred to the Department because of non-specific complaints, the etiology of which could not be explained otherwise. The ailments reported by the patients and their legal guardians typically included persistent joint and/or bone pain without a history of previous trauma, with concomitant swelling and/or fever or without.

The study was conducted according to the guidelines of the Declaration of Helsinki and approved by the Local Bioethics Committee of The Nicolaus Copernicus University, Collegium Medicum in Bydgoszcz, Poland (protocol code: KB401/2021, date of approval: 15 June 2021). All diagnostic images shown in this paper were fully anonymized; the legal guardians of the patients gave their written consent to publish the images.

The analysis included the documentation of outpatient and inpatient treatment and the results of imaging studies: conventional radiography, MRI, CT and ultrasound. The diagnostic process varied depending on the patient’s clinical condition, availability of imaging data from the referring center and legal guardians’ consent for further procedures. Hence, not all patients underwent all three types of diagnostic studies at our center. Similarly, bone biopsy was not carried out in some eligible cases given the lack of consent from the patient’s legal guardians.

All imaging studies were carried out at the Department of Imaging Diagnostics, Regional Children Hospital in Bydgoszcz. Conventional radiograms were obtained with an YSIO X-ray system (Siemens, Munich, Germany). CT scans were acquired with a 64-slice SOMATOM Definition AS scanner (Siemens, Munich, Germany). MRI was conducted with a 1.5 T Magnetom Essenza DOT device (Siemens, Munich, Germany). Follow-up MRIs were performed at various frequencies and time intervals (range: 3–24 months), depending on the patient’s clinical condition. The results of all imaging tests were interpreted by the first author (M.K.). Qualification for bone biopsy was based on the algorithm proposed by Taddio et al. [11].

The results were subjected to statistical analysis with Statistica 10 package (StatSoft, Tulsa, OK, USA). Statistical characteristics of continuous variables were presented as arithmetic means and ranges, and the distributions of discrete variables as numbers and percentages.

## 3. Results

**Figure 1:**
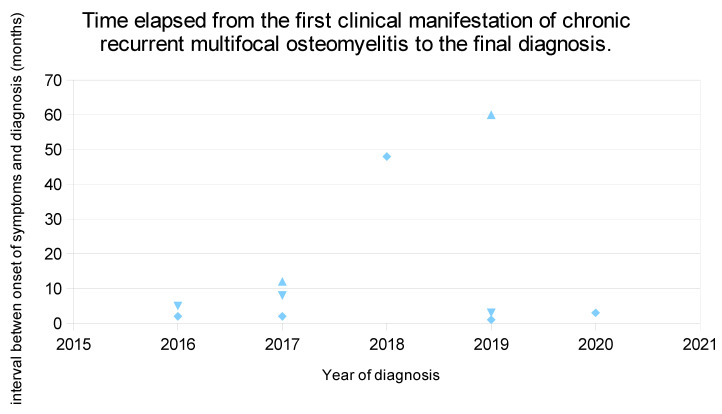
Time elapsed from the first clinical manifestation of chronic recurrent multifocal osteomyelitis to the final diagnosis.

Mean age at the first manifestation of the disease was 10 years and 7 months, with mean ages of male and female patients of 12 years (range 8–17) and 10 years (range 9–15), respectively. The mean time elapsed from the first clinical manifestation of the disease to the final diagnosis was 15.4 months (Figure 1). The mean age at diagnosis was 11 years and 10 months. Alternative diagnoses considered in the study patients included infectious osteitis, Ewing’s sarcoma, juvenile idiopathic arthritis and viral infection.

Unilateral lesions were found in eight patients (80%) and bilateral in two (20%). In three patients, the lesions were located in two anatomical areas (e.g., hip joint and tarsal joint), whereas, in the other seven patients, the disease was limited to only one anatomical region. Inflammatory lesions were most often located in the pelvic bones (33%) and lumbar spine (16%). Unifocal lesions were most commonly found in the tibia, fibula, clavicle, thoracic spine and sacrum (Table 2).

In 80% of the patients, the initial manifestation of the disease was bone pain. In 30% of the patients, conventional radiography demonstrated focal loss of bone density demarcated by an osteosclerotic zone; the radiograms of the other patients were normal (Table 3).

CT of the affected anatomical region was carried out in 30% of the patients (Table 3). CT scans demonstrated the presence of disseminated foci with a non-homogeneous osteolytic/osteosclerotic structure, with a massive loss of cortical layer and strong periosteal reaction. In the case of the thoracic and lumbar spine, CT showed a slight decrease in the height of vertebral bodies.

All patients with a presumptive diagnosis of CRMO underwent MRI (Table 3). Most patients presented with multifocal hypodense areas on T1-weighted images, with the enhancement of signal on T-weighted and STIR sequences. Additionally, the areas involved with the inflammatory process showed an intensive contrast enhancement. Furthermore, MRI demonstrated swelling of adjacent soft tissues and bone marrow edema. The abnormalities described above, in particular bone lesions, were found in all patients diagnosed with CRMO. Typical MRI findings in the thoracic and lumbar spine included the decreased height of vertebral bodies and bone marrow edema.

**Figure 2:**
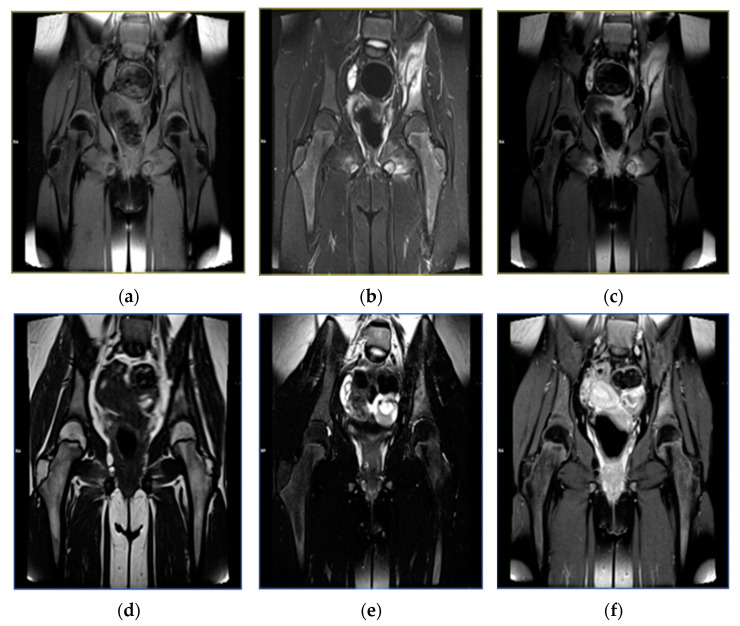
Magnetic resonance images in a 10-year-old female patient with inflammatory lesions around the left sacroiliac joint. Status before treatment: T1-weighted sequence (**a**), T2-weighted sequence (**b**), T1-weighted sequence with contrast enhancement (**c**). Status after a 19-month follow-up, with partial regression of the inflammatory lesions: T1-weighted sequence (**d**), T2-weighted sequence (**e**), T1-weighted sequence with contrast enhancement (**f**).

**Figure 3:**
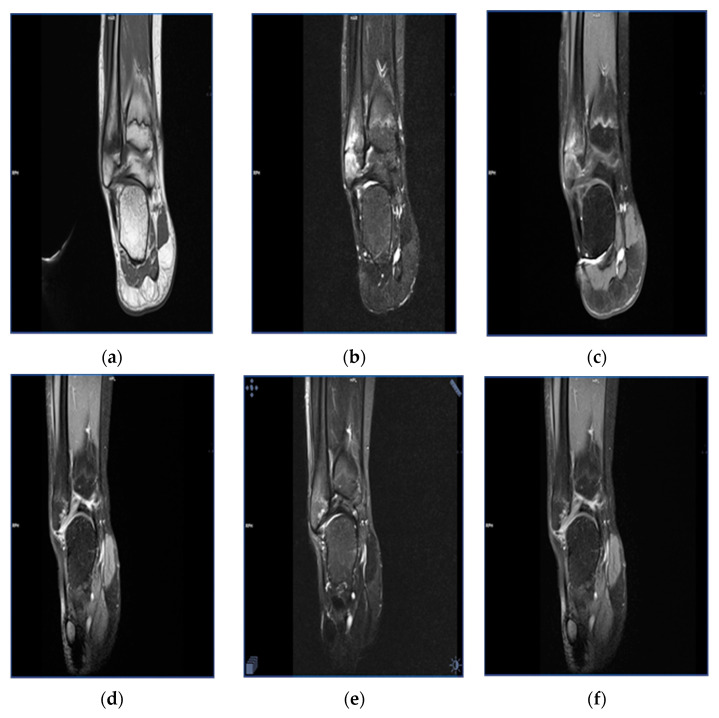
Magnetic resonance images in a 13-year-old female patient with bone marrow edema within the head of the fibula. Status before treatment: T1-weighted sequence (**a**), T2-weighted sequence (**b**), T1-weighted sequence with contrast enhancement (**c**). Status after a 15-month follow-up, with regression of the bone marrow edema: T1-weighted sequence (**d**), T2-weighted sequence (**e**), T1-weighted sequence with contrast enhancement (**f**).

**Figure 4:**
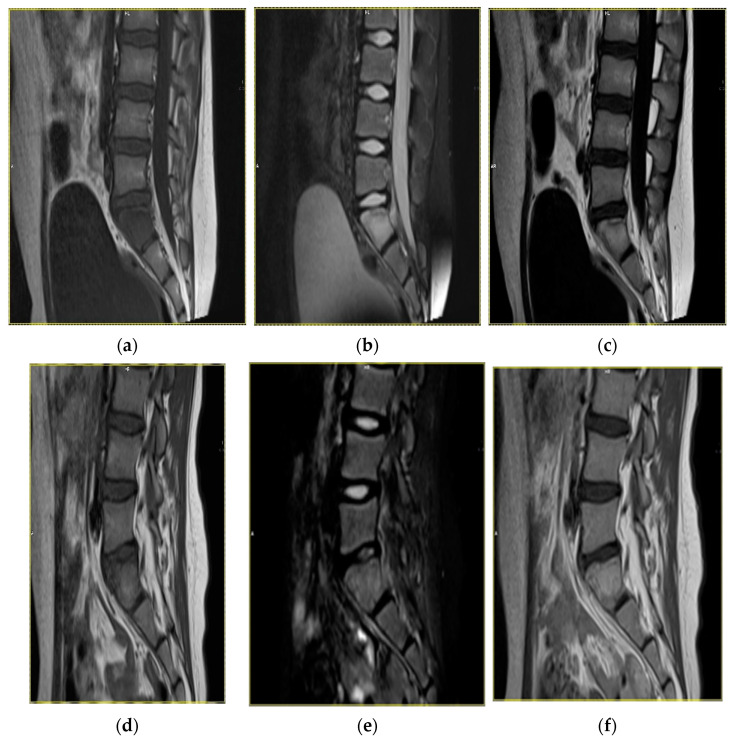
Magnetic resonance images in a 9-year-old female patient with inflammatory lesions within S1 sacral vertebra. Status before treatment: T1-weighted sequence (**a**), T2-weighted sequence (**b**), T1-weighted sequence with contrast enhancement (**c**). Status after an 8-month follow-up, with regression of the inflammatory lesions: T1-weighted sequence (**d**), T2-weighted sequence (**e**), T1-weighted sequence with contrast enhancement (**f**).

The duration of follow-up of the study patients varied between 3 months and 3 years. In 40% of the patients, both clinical symptoms and radiological abnormalities resolved completely, whereas another 50% of patients showed partial regression of clinical and radiological manifestations. In the remaining 10% of the patients, follow-up was too short to confirm progression/regression of the disease (Figure 2, Figure 3 and Figure 4).

## 4. Discussion

Due to a greater awareness of CRMO as a potential diagnosis in children with bone pain, the proper diagnosis may be reached earlier. In our study, the mean time elapsed from initial clinical manifestations of the disease until the ultimate diagnosis was 15.4 months; this is a relatively short period given that the lack of specific clinical, laboratory and imaging markers hinders the diagnostic process substantially.

A characteristic radiological feature of CRMO is the presence of osteolytic foci, frequently surrounded by a hyperdense bone structure. However, none of our patients presented with such lesions on baseline radiograms. This implicates a limited applicability of conventional radiography in the detection of osteolytic foci typical for CRMO. However, conventional radiography remains a useful tool in patients with suspected CRMO as it may identify other causes of skeletal pain to be considered on differential diagnosis. The CRMO-specific lesions could be visualized on CT. Our experiences indicate that CT has higher diagnostic value than conventional radiography in detecting CRMO. However, it needs to be stressed that although, due to technological advancements, the dose of ionizing radiation absorbed during CT is relatively low, it is still high enough to interfere with the development of pediatric patients.

MRI can visualize bone marrow edema within the involved bone, which typically manifests as hypointense areas on T1-weighted images and hyperintensity on T2-weighted and STIR images with strong contrast enhancement [16]. In this study, MRI proved to be the most accurate diagnostic option, demonstrating the presence of inflammatory lesions in all examined patients, even those with no abnormalities on ultrasound and conventional radiography. These observations imply that MRI plays a vital role in the evaluation of patients with presumed CRMO. The diagnostic value of the method seems to be higher compared with conventional radiography and CT.

MRI also remains the most accurate diagnostic option in patients with relapse. In 30% of patients included in this study, MRI demonstrated progression of primary foci as well as secondary foci, usually in the proximity of the primary foci or in the thoracic/lumbar spine. Clinically, such patients presented with pain of the involved anatomical area and/or impaired function of adjacent joints. Our study also confirmed the role of MRI as a gold diagnostic standard in patients with remission. In such patients, MRI showed a substantial decrease in the size of inflammatory foci within the bone, regression of bone marrow edema and resolution of lesions in adjacent soft tissues. Such radiological presentation correlated with partial or complete remission of clinical symptoms.

The results presented above confirm the leading role of MRI in establishing the correct diagnosis of CRMO, monitoring of treatment outcomes and detection of new lytic foci during relapse and progression of the disease. It needs to be stressed that, apart from being an accurate diagnostic option providing high-quality images, MRI is not associated with exposure to ionizing radiation and contrast agents are generally safe. Optimally, each patient with suspected CRMO should undergo a whole-body MRI as the gold standard [17]. This examination allows for the detection of all skeletal inflammatory foci, including clinically latent ones, in a relatively short time. State-of-the-art MR scanners can produce such high-quality whole-body images within 40 min. This will facilitate the planning of the therapeutic process and monitoring of its outcomes [18].

Notably, effective cooperation between pediatricians and radiologists will facilitate the proper diagnosis of CRMO without exposing patients to an invasive procedure, such as bone biopsy. No biopsy is needed if the presentation of the disease on imaging studies is typical [19], with multiple inflammatory foci [14,15]. Biopsy should be considered mainly in less typical cases with unifocal location and non-characteristic presentation on diagnostic imaging. In such cases, the lack of neoplastic cells in the biopsy specimen, and the presence of lymphocyte infiltrate, osteonecrosis, and/or bone marrow edema/fibrosis support the diagnosis of a non-bacterial inflammatory disease. Our series included three patients eligible for bone biopsy (patients no. 3, 5 and 7 in Table 3). In two of those cases (patients no. 3 and 7), the biopsy was not carried out given the lack of the legal guardians’ consent and the fact that the patients did not satisfy some criteria proposed by Taddio et al. [11]. In patient no. 5, with changes in the left clavicle, a bone biopsy was carried out at another center before referral to our department, which excluded malignant character of the process.

An unquestioned strength of this study stems from the fact that it included a relatively large group of patients with CRMO, a rare clinical condition. However, this study also has some limitations inherent to its observational character and associated primarily with the lack of standardization of the diagnostic process. Hence, the results presented herein should be considered as guidance for further research rather than a basis for ultimate conclusions regarding clinical practice. An important question which needs to be addressed in the nearest future is how to expedite the diagnostic process in patients with suspected CRMO. With no doubt, the process needs to be standardized through a set of evidence-based guidelines and should be minimally invasive yet cost-effective. Another direction of future research should be the identification of a highly accurate diagnostic marker of CRMO, whether an imaging finding or a laboratory parameter.

## 5. Conclusions

MRI remains the gold standard in the diagnosis and monitoring of CRMO. While bone biopsy has unquestioned value in cases were the imaging findings are atypical, its applicability can be limited in pediatric patients given frequent lack of parental consent for this procedure. This puts particular emphasis on effective cooperation between clinicians and specialists in diagnostic imaging during the course of the diagnostic process.

## Figures and Tables

**Table 1:** Clinical score for a diagnosis of CRMO according to Jansson et al. [10].

Risk Factor	Score
Normal blood cell count	13
Symmetric lesions	10
Lesions with marginal sclerosis	10
Normal body temperature	9
Vertebral, clavicular or sternal lesions	8
Radiologically proven lesions ≥ 2	7
CRP ≥ 1 mg/dL	6
Total clinical score	63

CRP, C-reactive-protein.

**Table 2:** Localization of bone lesions in the study patients.

Involved Region	Number of Foci (%)
Pelvis	4 (33%)
Tibia	1 (8%)
Fibula	1 (8%)
Small bones of the foot	1 (8%)
Clavicle	1 (8%)
Lumbar vertebral bodies	2 (16%)
Sacral vertebral bodies	1 (8%)
Thoracic vertebral bodies	1 (8%)

**Table 3:** Detailed results of baseline and follow-up imaging studies in CRMO patients.

Patient	Sex	Age at Diagnosis (Years)	Involved Region	X-ray	MRI	CT	USG	Follow-Up
1	F	10	Ilium, ischium rami, pubic bones	n/a	Inflammatory lesions in the left ilium, rami of both ischiums and both pubic bones	Disseminated focal lesions in pelvic bones with non-homogeneous osteolytic–osteosclerotic structure, with massive loss of cortical layer and severe periosteal reactions	n/a	3-year, regression
2	F	13	Fibular head and epiphysis, metatarsal bones I and II	Normal	Tarsal joints, calcaneus, metatarsal bone II	n/a	Normal	1.5-year, regression
3	M	8	Proximal epiphysis of the right tibia	Focal loss of bone density in the proximal epiphysis of the right tibia	Proximal epiphysis of the right tibia	Irregular osteolytic focus in the proximal epiphysis of the right tibia	Normal	3-year, regression
4	F	9	Sacral vertebra S1	n/a	Sacral vertebra S1, sacroiliac joint	Bone loss in sacral vertebra S1	n/a	9-month, partial regression
5	F	15	Left clavicle	n/a	Sternal segment of the clavicle	n/a	n/a	7-month, partial regression
6	F	14	Lumbar spine	n/a	Decreased anterosuperior edge of L3 vertebral body, bone marrow edema	n/a	n/a	3-year, partial regression
7	M	17	Right pubic body	Loss of bone density in the symphysis pubis	Right pubic body and superior ramus, bone marrow edema	n/a	Normal	8-month, complete regression
8	M	11	Lumbar spine	Normal	Decreased height of L4 and L5 vertebral bodies, bone marrow edema in inferior terminal laminas	n/a	n/a	3-month
9	F	9	Pubis, ischium and hip acetabulum on the right side	Loss of bone density in the right ischium	Pubis, ischium and hip acetabulum on the right side	Analogous findings as on MRI	Normal	1-year, partial regression
10	F	12	Hip joints	Normal	Hip joints	n/a	Normal	3-year, regression

F, female; M, male; n/a, not applicable.

## Data Availability

No new data were created or analyzed in this study. Data sharing is not applicable to this article.
